# Attachment, Perceived Partner Phubbing, and Retaliation: A Daily Diary Study

**DOI:** 10.1111/jopy.70012

**Published:** 2025-08-08

**Authors:** Katherine B. Carnelley, Claire M. Hart, Laura M. Vowels, Tessa Thejas Thomas

**Affiliations:** ^1^ University of Southampton Southampton UK; ^2^ FAmily and DevelOpment Research Center (FADO), Faculty of Social and Political Sciences, Institute of Psychology University of Lausanne Lausanne Switzerland

**Keywords:** attachment, daily diary, phubbing, relationship satisfaction, retaliation

## Abstract

**Objective:**

We conducted a diary study to investigate the role of adult attachment on responses to daily perceived partner phubbing in a sample of couple members (*N* = 196).

**Method:**

We focused on personal and relational well‐being as well as reactions to phubbing, retaliation reports, and motives as outcomes.

**Results:**

Results showed that on days when participants perceived their partner as phubbing them more, participants higher in attachment anxiety reported higher depressed mood and lower self‐esteem; however, their relationship satisfaction was not impacted. In response to partner phubbing, participants higher in attachment anxiety reported more resentment, curiosity, and retaliation in response to phubbing. When retaliating to phubbing, those higher in attachment anxiety did so to seek support and approval from others, whereas those higher in attachment avoidance did so to gain approval from others.

**Conclusions:**

Our findings contribute to the understanding of how adult attachment patterns influence people's responses to partners' phubbing and well‐being.

1

Phubbing is a portmanteau created from the words “phone” and “snubbing.” Perceived partner phubbing refers to the perception that a partner's phone use interferes with the face‐to‐face communication quality due to reduced partner attention (Roberts and David 2016). As technology becomes more prevalent in our daily interactions with others, it is important to understand how it may affect the quality of in‐person interactions, relationship functioning, and well‐being. Phubbing within romantic relationships has been shown to increase interpersonal conflict, resulting in lowered relationship satisfaction and poorer individual well‐being (Kelly et al. 2017). Researchers have begun to investigate how couple members respond to perceived phubbing and the extent to which couple members retaliate against perceived phubbing by mirroring this behavior. Thomas et al. (2022) found that when daily perceived partner phubbing was high, phubbees reported lower relationship satisfaction and greater feelings of anger, resentment, and retaliation. Furthermore, Thomas et al. examined the reasons for retaliation; participants reported using their phone in response to perceived phubbing for reasons of seeking revenge and support and approval from others. In the present paper, our aim was to build on past research by examining the role of adult attachment patterns in the responses to perceived partner phubbing. The present study used a daily diary to explore whether attachment anxiety and avoidance moderated the associations between perceived phubbing and daily reports of relational and personal well‐being, phubbing responses, and retaliation behaviors.

Attention and responsiveness to one's romantic partner positively influence relationship satisfaction (e.g., Arian‐Dinc and Gable 2023; McDaniel and Drouin 2019). Phubbing may interfere with the ability to attend to one's partner and interfere with the quality of time spent together according to the displacement hypothesis (Abbasi 2018). According to equity theory (Hatfield and Traupmann 1981), equal investment in a close relationship leads to better relationship satisfaction. If an individual perceives unequal investment in their relationship, they may feel distressed and try to obtain equity. Perceptions of partner phubbing may indicate unequal investment in the relationship. Research shows that when individuals perceive partner phubbing, they report more feelings of exclusion, lower intimacy, and lower partner responsiveness (Beukeboom and Pollmann 2021; McDaniel and Wesselmann 2021; Vanden Abeele et al. 2019). The phubbee may perceive that these negative evaluations are due to shortcomings in the relationship, resulting in lower relationship satisfaction (e.g., Chotpitayasunondh and Douglas 2018; McDaniel et al. 2018; Wang et al. 2017) or due to the phubbee's personal shortcomings, resulting in distress. For example, the phubbee may feel that their partner does not view them as important or interesting enough to hold their attention (Chotpitayasunondh and Douglas 2018), resulting in lower emotional well‐being and self‐esteem (e.g., McDaniel and Drouin 2019; Wang et al. 2017).

### 1.1 Partner Phubbing and Relationship Functioning

Perceptions of partner phubbing are associated with lower relationship functioning in cross‐sectional and diary studies (e.g., Courtright and Caplan 2020; McDaniel and Coyne 2016). Carnelley et al. (2023) extended this work and examined the extent to which perceptions of partner phubbing and enacted phubbing behavior reports predicted relationship functioning in a dyadic diary and 2 months later. They found that daily perceived phubbing was associated with lower relationship quality; however, the effects of perceived phubbing did not affect relationship functioning 2 months later. In addition, they found that enacted phubbing did not predict relationship quality daily or 2 months later. This suggests that perceptions are of prime importance.

### 1.2 Emotional Outcomes of Perceived Partner Phubbing

Past work has examined the effects of perceived partner phubbing on emotional outcomes and personal well‐being. Research suggests that perceived partner phubbing can be detrimental to phubbee's personal well‐being. Wang et al. (2017) found that couple members who perceived greater partner phubbing also reported greater depression. Research by Nazir (2017) examined other emotional responses to perceived phubbing and found that the majority of participants reported feeling annoyed (83%) and angry (67%). Similarly, Krasnova et al. (2016) found that perceived phubbing was associated with more jealousy and anger, and that perceived phubbing had a negative effect on relational cohesion indirectly via jealousy. Despite these largely negative effects, some participants reported feeling indifferent (38.1%) to perceived phubbing, suggesting phubbees may have mixed emotional responses.

Research from the wider technoference area (defined as intrusions and interruptions of digital devices during social interactions) shows similar largely negative effects of perceived technoference on personal well‐being. For example, McDaniel and Drouin (2019) explored the impact of technoference on couple interactions and individual well‐being in a daily diary study. They found that on days when technoference was perceived as higher, individuals reported lower interaction quality, increased conflict, and lower mood. These findings are consistent with those that show perceptions of partner phubbing are primarily linked to negative emotional outcomes.

### 1.3 Responses to Perceived Phubbing

These negative emotions may lead partners to retaliate against perceived partner phubbing by engaging with one's own smartphone in the presence of one's partner. There is a small amount of research that examines responses to perceived partner phubbing, including retaliation and motivations for retaliation. Some common responses to perceived phubbing are to ignore it (Kelly et al. 2017), to intervene, or to copy the partner's phubbing behavior (i.e., retaliate) (Krasnova et al. 2016). The motivations for each of these responses have not been investigated extensively. Partners may ignore partner phubbing as a positive passive response to avoid conflict, although it may negatively influence the phubbee's well‐being. In contrast, retaliation by imitating phubbing behavior may be a tit‐for‐tat strategy driven by revenge. Research shows that participants engage in phubbing despite acknowledging that it is annoying to others (Aagaard 2020). Thomas et al. (2022) examined responses to perceived partner phubbing in a daily diary study and found that on days when perceived partner phubbing was high, phubbees reported greater curiosity about the phubbing behavior, more resentment, and greater retaliation by using their smartphones. They were among the first to examine motivations for retaliation and found participants endorsed revenge, need for support, and need for approval from others. Understanding the behavioral responses of the phubbee and disentangling motivations behind why the phubbee may phub their partner is a focus of the current study; specifically, we aimed to examine the role of attachment patterns in these responses.

### 1.4 The Role of Adult Attachment

Herein, we take an attachment theoretical (Ainsworth et al. 1978; Bowlby 1969) approach to better understand couple members' responses to perceived partner phubbing. Attachment anxiety reflects a fear of abandonment by partners leading to a hypervigilance to threat cues due to experiences with inconsistently reliable caregivers (Mikulincer and Shaver 2016). Attachment anxiety is associated with higher perceived partner phubbing (Roberts and David 2022) and technoference (McDaniel et al. 2018). Attachment avoidance, in contrast, reflects discomfort with intimacy and interdependence and the use of deactivating strategies due to experiences with neglectful and rejecting caregivers. Research shows mixed findings regarding avoidance and perceived partner phubbing—some show no association (Carnelley et al. 2023; McDaniel et al. 2018) and some find a positive association for men but a negative association for women (Broning and Wartberg 2022).

Attachment theory and research suggest that not only will those high in attachment anxiety and/or avoidance perceive more partner phubbing, but they will react more negatively to it. Attachment anxiety and avoidance may exacerbate the negative effects of partner phubbing on relationship quality because they are likely to make negative attributions for ambiguous behaviors (Li et al. 2023) like phubbing. Those high in insecurity may view phubbing as an indicator of rejection. Although those high in attachment avoidance tend to turn attention away from relational information as a protective strategy, they tend to react with suppressed anger and high hostility to partner transgressions and are less forgiving (Mikulincer and Shaver 2005). Those with high attachment anxiety have low trait self‐esteem and unstable state self‐esteem (Brennan and Bosson 1998; Foster et al. 2007) that is more contingent on others' acceptance and approval (Knee et al. 2008; Park et al. 2004), and more reactive to negative interpersonal feedback (e.g., Hepper and Carnelley 2012). Those high (versus low) in attachment anxiety also respond with more depressed mood to negative partner behaviors (Mikulincer and Shaver 2005) and report lower relationship satisfaction and closeness on days with partner conflict (Campbell et al. 2005). Perceived partner phubbing may be particularly harmful for attachment‐anxious individuals' personal and relational wellbeing because it indicates a devaluing of the self. Consistent with this, Roberts and David (2016) found that cell phone conflict mediated the association between perceived partner phubbing and relationship satisfaction, particularly for those higher in attachment anxiety. In addition, David and Roberts (2021) found that manipulated partner phubbing (versus a control condition) led to more romantic jealousy for those higher (versus lower) in attachment anxiety. They also found that partner phubbing had an indirect effect on relationship satisfaction via jealousy for those higher, but not lower, in attachment anxiety. Furthermore, Mosley and Parker (2023) found (using an APIM model) in a sample of mixed‐gender couples that women (but not men) high in attachment anxiety reported lower relationship satisfaction when perceptions of partner phubbing were high; actors' avoidance did not moderate the effects of perceived partner phubbing on actors' relationship satisfaction. They also found that actors' perceived phubbing had a stronger negative effect on their partners' relationship quality when actors' attachment anxiety or avoidance was higher. Their results suggest that insecurity together with high perceived partner phubbing is particularly detrimental for couples' relationship quality.

Finally, Carnelley et al. (2023) extended this work and examined how attachment anxiety and avoidance were related to partners' daily phubbing and relationship quality using a dyadic diary method. They found no main effects of actor or partner's attachment anxiety or avoidance on daily relationship quality and no moderation effects of attachment anxiety or avoidance on daily perceived phubbing for the prediction of daily relationship quality. This work differs from other studies that typically rely on retrospective reports of typical partner phubbing behavior using a cross‐sectional design (e.g., Mosley and Parker 2023) and focuses on daily reports of perceived partner phubbing and relationship quality. These methodological differences may account for the different findings for the role of attachment. Attachment styles may have a greater effect on global perceptions than daily reports (which may be less susceptible to memory bias); this is consistent with Chang and Overall's (2022) research that found that those high in attachment anxiety reported lower recalled relationship quality than they did in weekly reports. The current study builds on past work by examining the effects of attachment on reactions to daily perceived partner phubbing for a variety of outcomes (e.g., personal well‐being, emotional reactions, and retaliation behaviors and motives).

### 1.5 The Present Study

We posed the following research questions and tested the following pre‐registered hypotheses with regard to the role of adult attachment patterns in the present study (Hypotheses 1, 2, 5 and 6 are replications; Hypotheses 3, 4, 7–10 are novel).

#### 1.5.1 Research Question 1

How does attachment style influence one's daily reports of relationship satisfaction and personal well‐being?

Individuals scoring higher (versus lower) in attachment avoidance or scoring higher (versus lower) in attachment anxiety will be more likely to report lower personal well‐being and lower relationship satisfaction (Hypothesis 1 and 2, respectively).

#### 1.5.2 Research Question 2

Does attachment style moderate the relationship between daily perceived phubbing and relationship satisfaction, anger/frustration, personal well‐being, and desire to retaliate?

On days when perceptions of phubbing are high, individuals with higher (vs. lower) attachment anxiety or higher (vs. lower) attachment avoidance will report lower personal well‐being (Hypotheses 3 and 4, respectively). On days when perceptions of phubbing are high, individuals scoring higher (versus lower) in attachment anxiety or scoring higher (versus lower) in attachment avoidance will be more likely to report lower relationship satisfaction (Hypotheses 5 and 6, respectively). On days when perceptions of phubbing are high, individuals scoring higher (versus lower) in attachment anxiety or scoring higher (versus lower) in attachment avoidance will be more likely to report higher levels of anger/frustration in response to perceived phubbing (Hypotheses 7 and 8, respectively). On days when perceptions of phubbing are high, individuals scoring higher (versus lower) in attachment anxiety or scoring higher (versus lower) in attachment avoidance will be more likely to engage in retaliatory behaviors (Hypotheses 9 and 10, respectively).

#### 1.5.3 Research Question 3

What is the association between attachment and motives for phubbing retaliation?

## 2 Method

### 2.1 Participants

We pre‐registered this study on the Open Science Framework with a target sample size of *N* = 150, which we calculated based on prior daily diary studies (e.g., McDaniel et al. 2020). We also conducted a sensitivity power analysis in *R* using the *simr* (Green 2019) package to estimate the actual power for 150 participants with 10 daily timepoints, with an effect size of β = 0.20 (small‐to‐medium effect size). The estimated power was 100% [95% CI = 99.63–100].

We recruited participants via online forums, social media (e.g., Facebook, Twitter, LinkedIn), and word of mouth. Our inclusion criteria required participants to be: (a) aged 18 or over, (b) currently in a romantic relationship of at least 6 months duration, and (c) living with their current partner. Participants were required to complete the baseline survey and at least one diary survey. The initial sample included 269 participants who had completed the baseline survey; 73 participants did not continue the study beyond the baseline, or their daily diary entries could not be matched with their ID at baseline and were thus not included in the analyses.

The final sample consisted of a total of 196 participants^1^ (*Mᵃᵍᵉ* = 36.39, SD = 12.02). The majority of participants identified as female (*n* = 144), with 50 identifying as male, 1 as non‐binary, and 1 preferred not to say. When asked to describe their sexual orientation, most (*n* = 168) identified as straight; the rest were as follows: bisexual (*n* = 8), lesbian (*n* = 14), gay (*n* = 3), and other (*n* = 3). Participants reported on their current relationship status. Dating was the least selected option (*n* = 5) with the majority of the sample either married (*n* = 100) or in a committed relationship (*n* = 90); 49.5% had children whereas 50.5% did not. The average relationship length was approximately 11.4 years (SD = 9.91).

Most participants were either employed full time (53.6%) or students (11.2%). Several participants selected “other” for occupation (9.2%), for reasons such as working two jobs or living with a disability. The remaining few stated they were employed part time (9.7%) or homemakers (3.6%).

We collected data from February 2021 to March 2023.

### 2.2 Procedure

The study was ethically approved by the Faculty Ethics Committee at (anonymised for review). Participants first read the study information sheet and provided informed consent.

We advertised the study as a daily diary about “Mobile Phone Use in Romantic Relationships.” Participants were asked to complete one baseline diary followed by nine short daily diaries. The daily diaries were presented online via Qualtrics. Participants completed an average of 7.91 days, including baseline. The study took about an hour to complete across 10 days (5 min/day). Baseline measures (Day 1) included demographic questions (e.g., gender, sexual orientation, employment, relationship length), adult attachment and narcissistic trait measures^2^. At Baseline, participants that did not meet the inclusion criteria were unable to continue the study. The daily diaries measured perceived partner phubbing, responses to partner phubbing, relationship satisfaction, personal well‐being (i.e., depressed mood, anxious mood, self‐esteem), and anger/frustration. The daily measures were randomized to prevent order effects.

After completing the baseline measures, participants were either entered into a prize draw to win 2 of 5 £50 Amazon vouchers and were told that each daily diary completed would result in an additional five raffle tickets, or they were paid £6 for completion of all 10 sessions (£1.50 for the baseline survey and 50p for each diary session). When participants had completed the study, they were debriefed and thanked.

### 2.3 Measures

#### 2.3.1 Daily Perceived Phubbing

We measured daily perceptions of partner phubbing with four of the original nine items, adapted from the Pphubbing Scale (Roberts and David 2016): “Today, my partner placed his/her mobile phone where they could see it when we were together”, “Today, my partner glanced at his/her mobile phone when talking to me,” “Today, when my partner's phone rang or beeped, they pulled it out even if we were in the middle of a conversation,” and “Today, when my partner and I were together, my partner's mobile phone use interfered with our interactions.” Participants reported their partner's mobile phone use, on a 5‐point Likert scale (1 = *not at all*, 5 = *a great deal*).

#### 2.3.2 Daily Relationship Satisfaction

We measured daily romantic relationship satisfaction using the satisfaction subscale of the Perceived Relationship Quality Component Inventory (PRQC; Fletcher et al. 2000). Participants rated three items on a 7‐point Likert scale, ranging from 1 (*not at all*) to 7 (*extremely*).

#### 2.3.3 Daily Self‐Esteem

We measured daily self‐esteem with the Single‐Item Self‐Esteem Scale (SISE; Robins et al. 2001). Participants reported how they felt that day: “I have high self‐esteem”, on a 7‐point Likert scale (1 = *not very true of me*, 7 = *very true of me*).

#### 2.3.4 Daily Depressed/Anxious Mood

We assessed daily reports of depressed and anxious mood with the 4‐item Patient Health Questionnaire (PHQ‐4; Kroenke et al. 2009). The original instructions were modified to focus on how participants felt that day, for example, “Today, have you been bothered by any of these problems?”. Items measured anxious mood (“Feeling nervous, anxious, or on edge”, “Not being able to stop or control worrying”) and depressed mood (“Feeling down, depressed, or hopeless”, “Little interest or pleasure in doing things”). Participants rated items on a 4‐point Likert scale (1 = *not at all*, 4 = *a great deal*).

#### 2.3.5 Daily Anger/Frustration

We created three items to assess anger/Frustration: “Today, I felt angry,” “Today, I felt irritated,” “Today, I felt annoyed.” Participants reported daily how they felt on a 5‐point Likert scale, ranging from 1 (*not at all*) to 5 (*a great deal*).

#### 2.3.6 Daily Responses to Being Phubbed

If participants reported partner phubbing, we asked them six questions regarding how they responded to the phubbing: “I told them I was not happy” (conflict), “I asked them what they were looking at” (curiosity), “I argued with them about their phone use” (conflict), “I felt resentful about their phone use” (resentment), “I ignored their phone use” (ignored) and “I picked up my own phone and used it” (retaliation). Participants rated these items on a 9‐point Likert scale (1 = *not at all*, 5 = *a moderate amount*, 9 = *a great deal*). Because each item assessed a different construct, each was analyzed separately. Because the following two items overlapped conceptually, we computed an average to indicate conflict in response to phubbing: “I told them I was not happy” and “I argued with them about their phone use.”

#### 2.3.7 Daily Motivations for Retaliation

If a participant reported on the daily responses that they retaliated when they were phubbed on this item (indicated by a score greater than 1 on this item, “I picked up my own phone and used it”), they were presented with an additional four items. Participants were rated on an 8‐point Likert scale (1 = *strongly disagree*, 8 = *strongly agree*) why they chose to pick up their own phone and use it. Items included: “To get back at my partner,” “I was bored,” “To seek support from others,” “To seek approval from others.” We examined each item separately in the statistical analysis.

#### 2.3.8 Adult Attachment

Participants completed the ECR‐12 (Lafontaine et al. 2016) to assess general romantic attachment anxiety and avoidance. Participants rated on an 8‐point scale (1 = *Do not agree at all*) to 8 (*Agree completely*) their agreement with 12 items.

### 2.4 Data Analysis

The daily diary data were nested, i.e., days (level 1) nested within individuals (level 2); therefore, we analyzed them using hierarchical linear modeling (HLM) with the *lme4* package in R (R Core Team 2021). Level 1 variables represented within‐person variations (i.e., daily perceived phubbing effects on daily response behavior, daily relationship satisfaction, daily anger/frustration, daily depressed mood, daily anxious mood, daily self‐esteem). This allowed us to examine daily fluctuations in emotions and behavior based on perceived partner phubbing. Time (scaled to start at 0) was factored in as a covariate throughout. Level 2 variables were attachment anxiety and avoidance. We also included an interaction between daily perceived phubbing and attachment anxiety and avoidance, respectively. We used Restricted maximum likelihood (REML) to handle missing data. There is no clear agreement on appropriate measures of effect size in multilevel modeling (Peugh 2010); therefore, we report marginal and conditional R squared values, as suggested by Nakagawa and Schielzeth (2012). We used the *multilevel.reliability* function from the *psych* package to estimate the reliability of the time‐varying variables.

## 3 Results

During data cleaning, duplicate participant surveys pertaining to 1 day were removed. Checks for normality revealed skewness for certain variables, including conflict, motivation for retaliation: revenge, motivation for retaliation: support, and motivation for retaliation: approval. These variables were log transformed prior to analysis. Daily phubbing was person‐mean centered to test for within‐person effects. Table 1 displays descriptive statistics for daily data across 9 days. We report the reliability of the average of all ratings across all items and times (fixed time effects) in Table 1, but other forms of reliability are available on the OSF project page in the results file for interested readers. For attachment anxiety and avoidance, we report Cronbach's alphas. Several multilevel models were conducted, using the *lme4 R* package, to explore the nested data at a within‐person level (level 1). All models included both random intercepts and random slopes. All Data S1, including data and R code, can be found on the OSF project page^3^: https://osf.io/ts6gw/?view_only=f4f774dbc6bc4de78eb7677c65dc4ab6.

**TABLE 1:** Descriptive statistics for all baseline and daily measures.

Variable	M	SD	Skewness	Kurtosis	Reliability
Level 1					
Perceived partner phubbing	2.45	0.80	0.43	0.03	0.96
Response: Curiosity	3.12	2.40	0.87	−0.52	
Response: Resentment	2.14	2.02	1.87	2.51	
Response: Ignored	5.74	2.71	−0.50	−1.07	
Response: Conflict^a^	1.60	1.29	2.50	6.37	
Response: Retaliation	4.64	2.65	−0.06	−1.34	
Motivation for retaliation: Revenge	1.95	1.67	1.77	1.77	
Motivation for retaliation: Boredom	5.43	1.97	−0.69	−0.24	
Motivation for retaliation: Support^a^	1.50	1.30	3.32	11.24	
Motivation for retaliation: Approval^a^	1.38	1.02	3.67	15.32	
Relationship satisfaction	5.70	1.25	−1.10	1.10	1.00
Anger/frustration	1.72	0.84	1.51	2.20	0.98
Self‐esteem	4.12	1.74	−0.18	−0.89	
Anxious mood	1.68	0.80	1.19	0.68	0.98
Depressed mood	1.64	0.78	1.23	0.87	0.98
Level 2					
Attachment anxiety	5.65	1.13	−0.31	−0.57	0.87
Attachment avoidance	4.65	0.92	−0.79	1.06	0.88

^a^ Log transformed for analyses.

### 3.1 Daily Relationship Satisfaction and Individual Well‐Being

**FIGURE 1:**
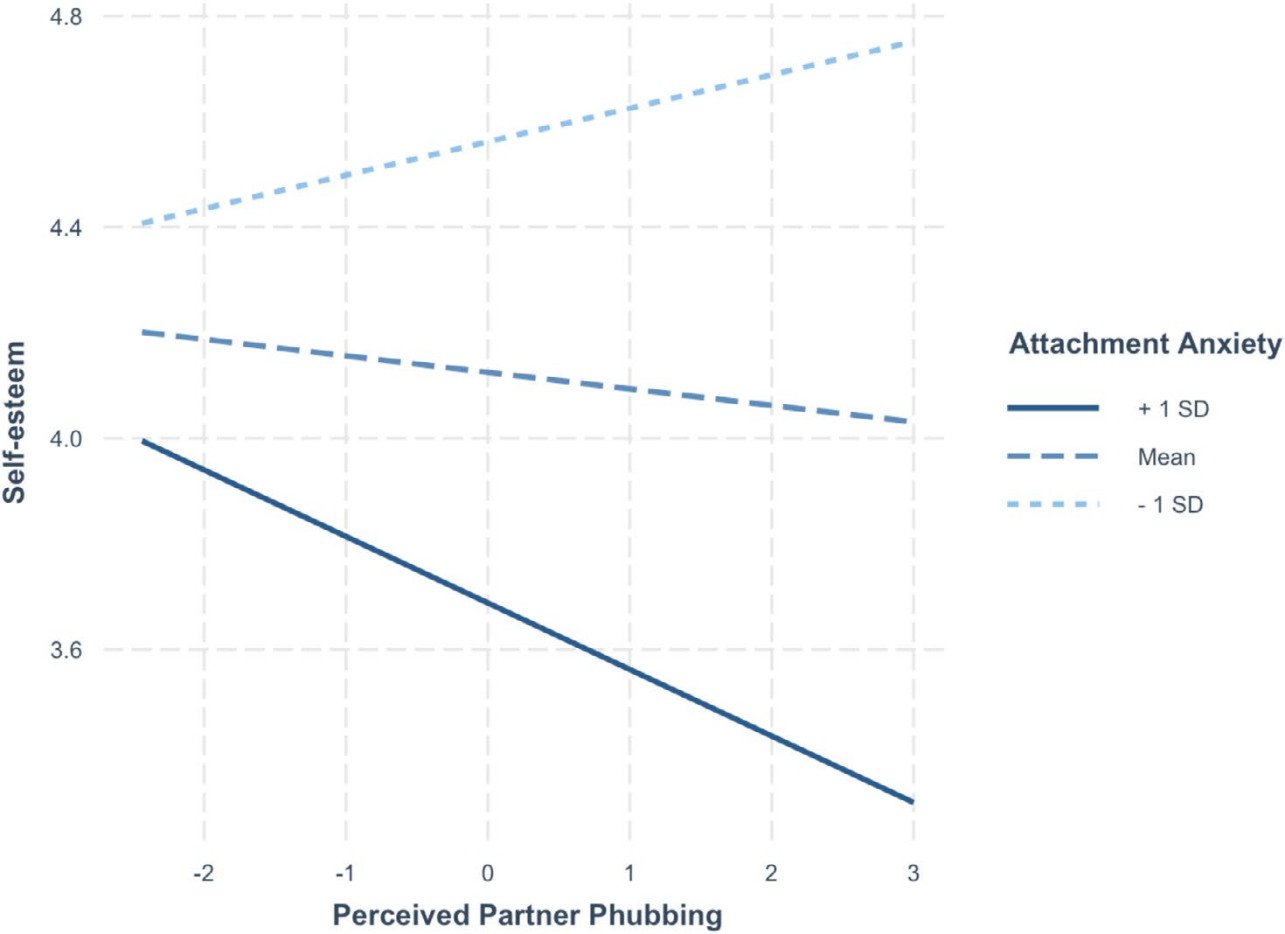
Slopes for moderation by attachment anxiety of effect of partner phubbing on self‐esteem.

**FIGURE 2:**
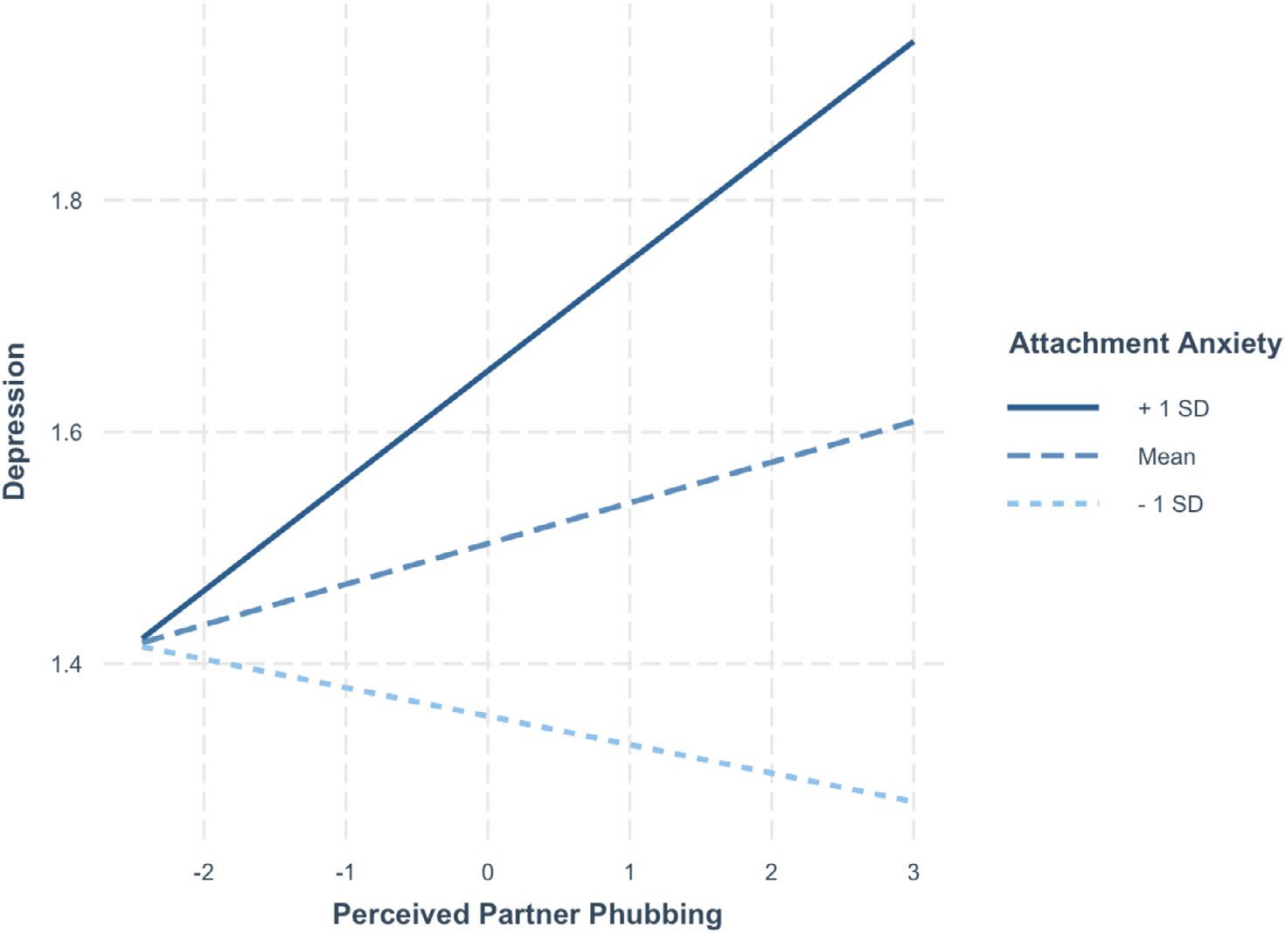
Slopes for moderation by attachment anxiety of effect of partner phubbing on depression.

See Table 2 for the full results for the relationship and individual well‐being variables. On days when participants reported higher levels of perceived partner phubbing, they also reported lower relationship satisfaction (*p* < 0.001), higher anxious mood (*p* = 0.009), and higher anger (*p* < 0.001).^4^ Participants higher in attachment anxiety reported lower self‐esteem (*p* = 0.012), higher anxious (*p* < 0.001) and depressed mood (*p* < 0.001), and higher anger (*p* < 0.001) on average. Participants higher in attachment avoidance reported lower relationship satisfaction (*p* < 0.001), lower self‐esteem (*p* < 0.001), and higher anger (*p* = 0.010) on average. Two interaction effects were significant: attachment anxiety and perceived partner phubbing significantly interacted to predict self‐esteem (*p* = 0.003) and depression (*p* = 0.002). The simple slopes analyses showed that on days when they perceived their partner as phubbing them more, participants higher in attachment anxiety (1 SD above the mean) reported significantly lower self‐esteem (*B* = −0.12, *t* = −2.88, *p* = 0.004) and higher depressed mood (*B* = 0.09, *t* = 3.63, *p* < 0.001) (see Figures 1 and 2). By contrast, participants lower in attachment anxiety (1 SD below the mean) did not report significantly lower self‐esteem (*B* = 0.06, *t* = 1.22, *p* = 0.222) or higher depressed mood (*B* = 0.03, *t* = −0.78, *p* = 0.434). No other variables were significant.

**TABLE 2:** Results of perceived partner phubbing and attachment anxiety and avoidance predicting relationship and individual well‐being.

Relationship satisfaction				Self‐esteem			Anxious mood			Depressed mood			Anger/frustration		
Predictors	Estimates	CI	*p*	Estimates	CI	*p*	Estimates	CI	*p*	Estimates	CI	*p*	Estimates	CI	*p*
Intercept	5.82	5.68–5.97	**< 0.001**	4.07	3.85–4.29	**< 0.001**	1.65	1.56–1.73	**< 0.001**	1.57	1.47–1.66	**< 0.001**	1.65	1.56–1.74	**< 0.001**
Partner phubbing	−0.17	−0.23 to −0.11	**< 0.001**	−0.03	−0.10 to 0.04	0.382	0.06	0.02–0.10	**0.009**	0.04	−0.01 to 0.08	0.101	0.17	0.11–0.22	**< 0.001**
Attachment anxiety	−0.07	−0.15 to 0.01	0.107	−0.26	−0.38 to −0.13	**< 0.001**	0.13	0.08–0.17	**< 0.001**	0.09	0.04–0.13	**< 0.001**	0.09	0.05–0.14	**< 0.001**
Attachment avoidance	−0.44	−0.55 to −0.34	**< 0.001**	−0.21	−0.36 to −0.05	**0.012**	0.03	−0.02 to −0.09	0.225	0.04	−0.02 to −0.09	0.216	0.07	0.02–0.13	**0.010**
Time	−0.01	−0.03 to −0.01	0.407	0.01	−0.01 to 0.03	0.165	−0.02	−0.03 to −0.01	**0.001**	−0.02	−0.03 to −0.00	**0.006**	−0.01	−0.03 to −0.00	**0.044**
Phubbing^*^ attachment anxiety	−0.01	−0.04 to 0.02	0.627	−0.06	−0.09 to −0.02	**0.003**	0.02	−0.01 to 0.04	0.125	0.04	0.01–0.06	**0.002**	−0.03	−0.06 to 0.00	0.080
Phubbing^*^ attachment avoidance	−0.00	−0.05 to 0.04	0.874	0.00	−0.05 to 0.06	0.917	0.01	−0.03 to 0.04	0.735	−0.00	−0.03 to 0.03	0.965	0.02	−0.02 to 0.07	0.263
**Random effects**															
*σ* ^2^	0.43			0.59			0.25			0.21			0.37		
τ_00_	0.92_ParticipantID_			2.32_ParticipantID_			0.28_ParticipantID_			0.37_ParticipantID_			0.26_ParticipantID_		
τ_11_	0.01_ParticipantID.time_			0.01_ParticipantID.time_			0.00_ParticipantID.time_			0.00_ParticipantID.time_			0.00_ParticipantID.time_		
ρ_01_	−0.07_ParticipantID_			0.07_ParticipantID_			−0.30_ParticipantID_			−0.49_ParticipantID_			−0.35_ParticipantID_		
ICC	0.71			0.81			0.52			0.59			0.40		
N	210_ParticipantID_			209_ParticipantID_			210_ParticipantID_			210_ParticipantID_			210_ParticipantID_		
Observations	1518			1518			1521			1506			1516		
Marginal *R* ^2^/Conditional *R* ^2^	0.204/0.767			0.079/0.827			0.094/0.564			0.053/0.616			0.071/0.439		

*Note:* Bold values denote statistically significant results.

### 3.2 Daily Responses to Being Phubbed

**FIGURE 3:**
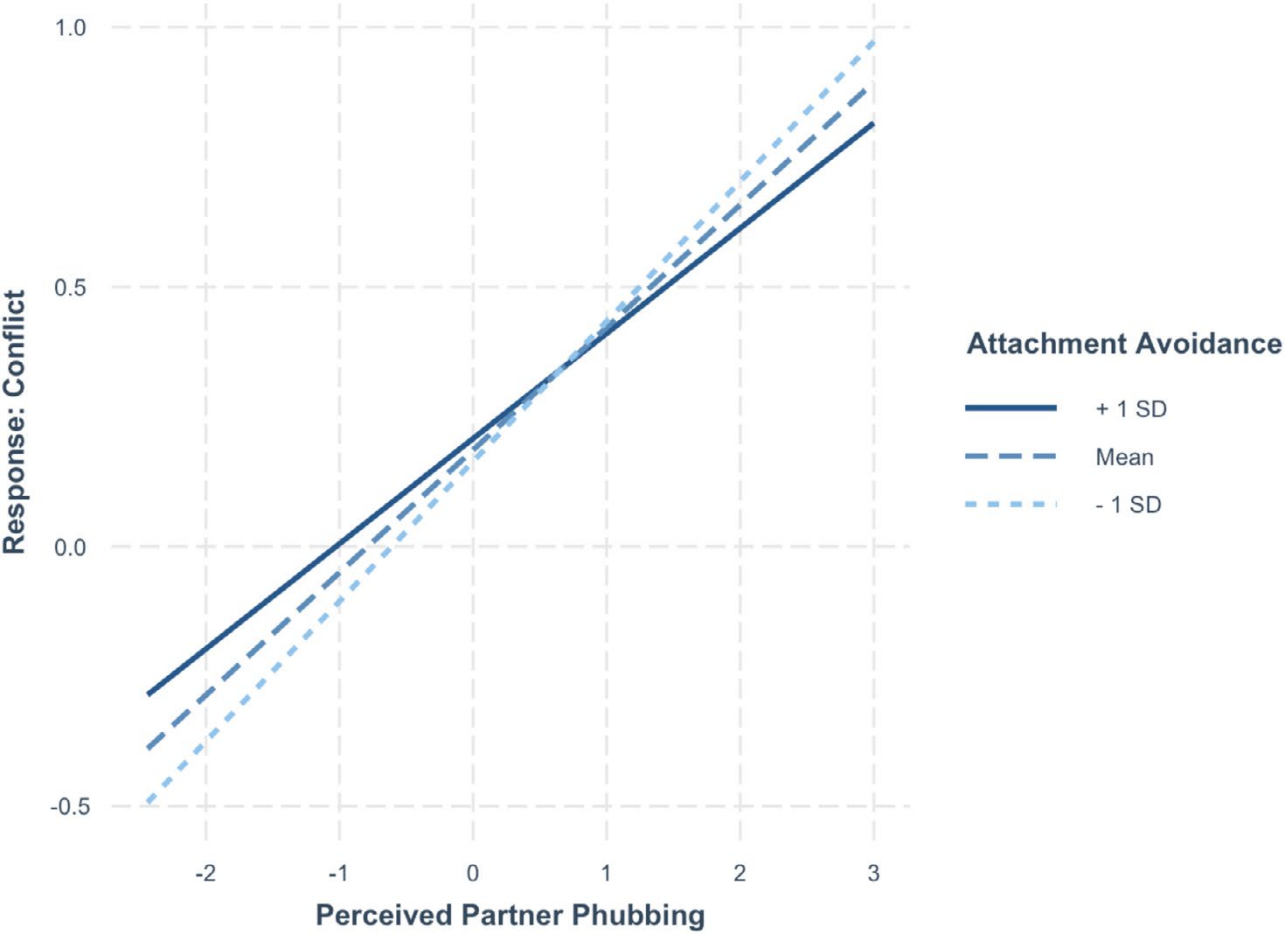
Slopes for moderation by attachment avoidance of effect of partner phubbing on conflict.

Several responses towards perceived partner phubbing were analyzed: curiosity towards a partner's phone use, resentment, ignoring partner phubbing, conflict towards partner phubbing, and tit‐for‐tat retaliation towards partner phubbing. See Table 3 for the full results for the daily responses to being phubbed. On days when participants reported higher levels of perceived partner phubbing, they also reported significantly greater curiosity (*p* < 0.001), resentment (*p* < 0.001), conflict (*p* < 0.001), and retaliation (*p* < 0.001). In contrast, they were significantly less likely to feel ignored (*p* = 0.004).^5^ Individuals higher (versus lower) in attachment anxiety also reported significantly higher curiosity (*p* = 0.029), resentment (*p* = 0.004), and retaliation (*p* = 0.008). There was only one significant interaction between attachment avoidance and perceived partner phubbing in predicting conflict (*p* = 0.045). The simple slopes analyses showed that on days when they perceived their partner as phubbing them more (versus less), participants higher in attachment avoidance (1 SD above the mean) reported significantly higher conflict (*B* = 0.20, *t* = 8.54, *p* < 0.001) (see Figure 3). However, on days when they perceived more phubbing, participants with lower attachment avoidance (1 SD below the mean) reported even higher levels of conflict (*B* = 0.27, *t* = 11.19, *p* < 0.001). No other variables were significant.

**TABLE 3:** Results of perceived partner phubbing and attachment anxiety and avoidance predicting daily responses to being phubbed.

Curiosity				Resentment			Ignored			Conflict			Retaliation		
Predictors	Estimates	CI	*p*	Estimates	CI	*p*	Estimates	CI	*p*	Estimates	CI	*p*	Estimates	CI	*p*
Intercept	2.45	2.19–2.72	**< 0.001**	1.68	1.46–1.90	**< 0.001**	5.81	5.47–6.14	**< 0.001**	0.17	0.11–0.23	**< 0.001**	3.82	3.51–4.13	**< 0.001**
Partner phubbing	1.20	1.05–1.35	**< 0.001**	0.99	0.87– 1.10	**< 0.001**	−0.37	−0.63 to −0.12	**0.004**	0.24	0.20–0.27	**< 0.001**	1.01	0.84–1.19	**< 0.001**
Attachment anxiety	0.15	0.01–0.28	**0.029**	0.16	0.05–0.27	**0.004**	−0.06	−0.23 to 0.11	0.486	0.03	−0.00 to 0.06	0.063	0.23	0.06–0.39	**0.008**
Attachment avoidance	−0.05	−0.21 to 0.12	0.567	0.09	−0.05 to 0.22	0.196	−0.02	−0.23 to 0.19	0.863	0.02	−0.02 to 0.05	0.381	0.05	−0.15 to 0.26	0.617
Time	−0.03	−0.07 to 0.00	0.088	−0.00	−0.03 to 0.03	0.970	−0.08	−0.14 to −0.01	**0.016**	0.00	−0.00 to 0.01	0.338	−0.13	−0.18 to −0.09	**< 0.001**
Phubbing * attachment anxiety	−0.01	−0.10 to 0.07	0.774	0.06	−0.00 to 0.13	0.053	−0.08	−0.21 to 0.06	0.280	−0.00	−0.02 to 0.01	0.597	−0.02	−0.12 to 0.07	0.651
Phubbing^*^ attachment avoidance	−0.09	−0.20 to 0.03	0.130	0.01	−0.08 to 0.10	0.805	−0.16	−0.35 to 0.03	0.098	−0.03	−0.05 to −0.00	**0.045**	0.01	−0.12 to 0.14	0.867
**Random effects**															
*σ* ^2^	2.05			1.16			5.64			0.09			2.59		
τ_00_	2.61_ParticipantID_			1.94_ParticipantID_			2.68_ParticipantID_			0.14_ParticipantID_			3.57_ParticipantID_		
τ_11_	0.01_ParticipantID.time0_			0.01_ParticipantID.time0_			0.04_ParticipantID.time0_			0.00_ParticipantID.time0_			0.03_ParticipantID.time0_		
ρ_01_	−0.45_ParticipantID_			−0.48_ParticipantID_			0.08_ParticipantID_			−0.43_ParticipantID_			−0.13_ParticipantID_		
ICC	0.53			0.59			0.39			0.58			0.60		
*N*	210_ParticipantID_			209_ParticipantID_			210_ParticipantID_			210_ParticipantID_			210_ParticipantID_		
Observations	1230			1227			1229			1228			1230		
Marginal *R* ^2^/Conditional *R* ^2^	0.117/0.584			0.135/0.643			0.012/0.399			0.089/0.619			0.095/0.637		

*Note:* Bold values denote statistically significant results.

### 3.3 Daily Motivations for Retaliation

**FIGURE 4:**
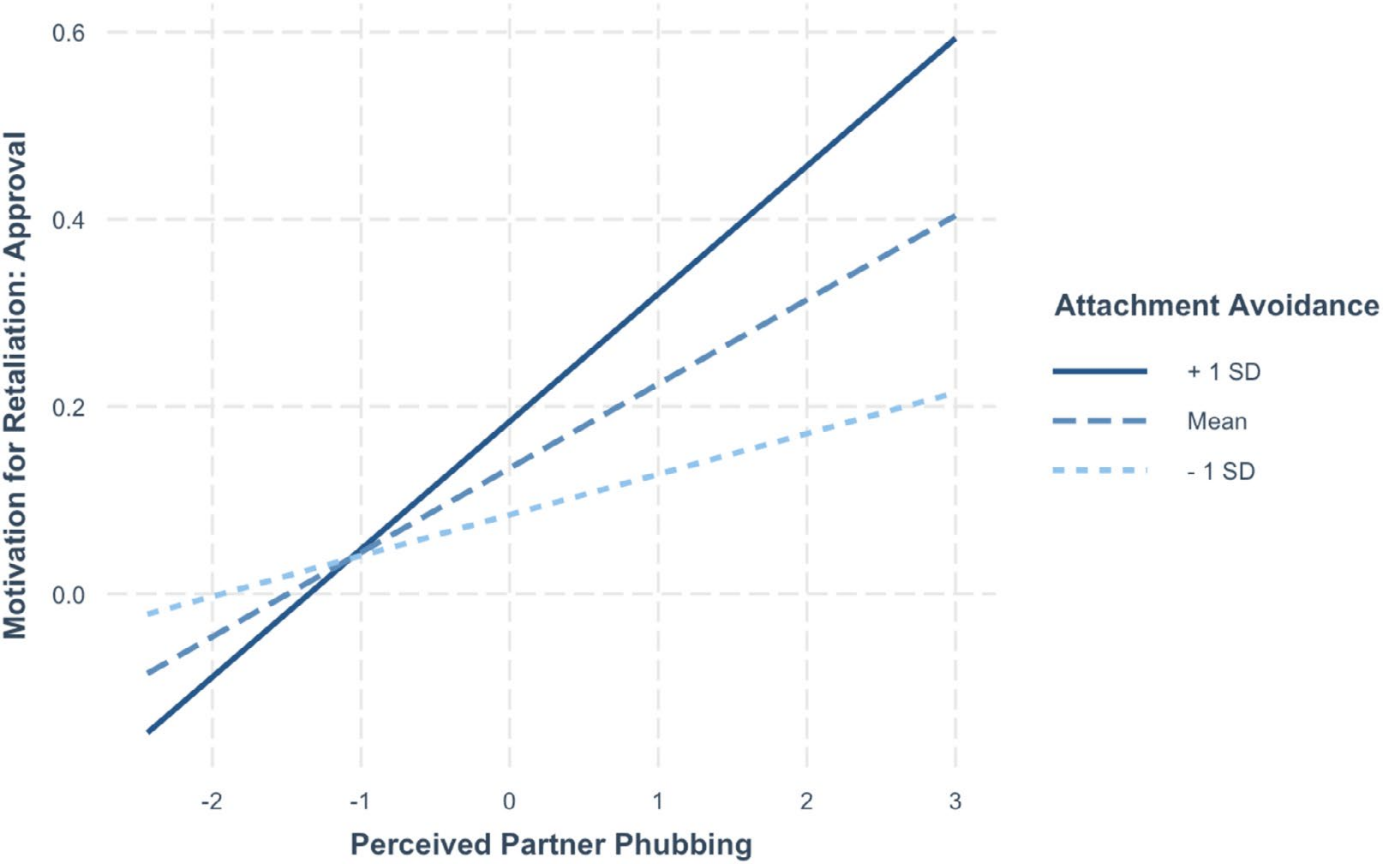
Slopes for moderation by avoidance of effect of partner phubbing on approval seeking.

Alongside understanding how phubbees responded to partner phubbing, we assessed why one may engage in tit‐for‐tat retaliation behavior (i.e., picking up one's own phone and using it as a response to perceived partner phubbing). This research question aimed to address why phubbees retaliated against their partner. Four possible motives were: revenge, boredom, need for support, and need for approval. See Table 4 for the full results for daily motivations for retaliation. On days when participants reported higher levels of perceived partner phubbing, they also reported significantly higher agreement with all of the motivations: revenge (*p* < 0.001), boredom (*p* < 0.001), need for support (*p* = 0.017), and need for approval (*p* < 0.001).^6^ Individuals higher in attachment anxiety also reported significantly higher need for support (*p* = 0.014) and need for approval (*p* = 0.010). There was only one significant interaction between attachment avoidance and perceived partner phubbing in predicting need for approval (*p* = 0.037). The simple slopes analyses showed that on days when they perceived their partner as phubbing them more, participants higher in attachment avoidance (1 SD above the mean) reported significantly higher need for approval (*B* = 0.14, *t* = 4.60, *p* < 0.001) (see Figure 4). Participants lower in attachment avoidance (1 SD below the mean) did not report a significantly higher need for approval (*B* = 0.05, *t* = 1.50, *p* = 0.134). No other variables were significant.

**TABLE 4:** Results of perceived partner phubbing and attachment anxiety and avoidance predicting daily motivations for retaliation.

Revenge				Boredom			Need for support			Need for approval		
Predictors	Estimates	CI	*p*	Estimates	CI	*p*	Estimates	CI	*p*	Estimates	CI	*p*
Intercept	0.29	0.21–0.36	**< 0.001**	4.97	4.67–5.26	**< 0.001**	0.21	0.14–0.28	**< 0.001**	0.15	0.09–0.21	**< 0.001**
Partner phubbing	0.19	0.14–0.24	**< 0.001**	0.50	0.30–0.70	**< 0.001**	0.06	0.01–0.10	**0.017**	0.09	0.05–0.13	**< 0.001**
Attachment anxiety	0.04	−0.00 to 0.08	0.058	0.15	−0.01 to 0.30	0.069	0.04	0.01–0.08	**0.014**	0.04	0.01–0.07	**0.010**
Attachment avoidance	0.04	−0.01 to 0.09	0.091	0.07	−0.13 to 0.27	0.481	0.03	−0.02 to 0.07	0.208	0.04	−0.00 to 0.08	0.069
Time	−0.02	−0.03 to −0.01	**0.001**	−0.09	−0.14 to −0.04	**0.001**	−0.01	−0.02 to 0.00	0.265	−0.00	−0.01 to 0.01	0.459
Phubbing * attachement anxiety	0.02	−0.01 to 0.05	0.257	0.09	−0.02 to 0.20	0.120	0.02	−0.00 to 0.05	0.083	0.00	−0.02 to 0.03	0.760
Phubbing^*^ attachment avoidance	−0.01	−0.06 to 0.03	0.505	−0.16	−0.32 to 0.00	0.050	0.00	−0.03 to 0.04	0.852	0.03	0.00 to 0.07	**0.037**
**Random effects**												
*σ* ^2^	0.14			1.85			0.11			0.08		
τ_00_	0.15_ParticipantID_			2.59_ParticipantID_			0.12_ParticipantID_			0.10_ParticipantID_		
τ_11_				0.03_ParticipantID.time0_			0.00_ParticipantID.time0_			0.00_ParticipantID.time0_		
ρ_01_				−0.25_ParticipantID_			−0.23_ParticipantID_			−0.23_ParticipantID_		
ICC	0.53			0.59			0.51			0.59		
N	185_ParticipantID_			185_ParticipantID_			184_ParticipantID_			184_ParticipantID_		
Observations	740			741			738			738		
Marginal *R* ^2^/Conditional *R* ^2^	0.082/0.567			0.058/0.611			0.044/0.533			0.062/0.612		

*Note:* Bold values denote statistically significant results.

## 4 Discussion

### 4.1 Daily Relationship Satisfaction and Individual Well‐Being

Results showed that as expected (Hypothesis 1) participants higher in attachment anxiety reported lower self‐esteem, higher anxious and depressed mood, and more anger on average, but not lower relationship satisfaction on average (contrary to Hypothesis 2). Also as expected (Hypothesis 1 and 2), participants higher in attachment avoidance reported lower relationship satisfaction, lower self‐esteem, and higher anger on average. This is generally consistent with past research that shows attachment anxiety is associated with indicators of a negative self‐model and distress, and avoidance is associated with negative models of others and poor close relationship quality (Bartholomew and Horowitz 1991; Mikulincer and Shaver 2016).

Importantly, attachment anxiety moderated the effects of daily perceived partner phubbing to predict self‐esteem and depression. Participants higher in attachment anxiety reported lower self‐esteem and a higher depressed mood on days when they perceived their partner as phubbing them more. These novel findings confirm Hypothesis 3 and demonstrate that perceived partner phubbing can have detrimental effects for personal well‐being for those high in attachment anxiety. This is consistent with past work that demonstrates that their self‐worth is contingent on relational sources and interpersonal feedback (e.g., Hepper and Carnelley 2012). Contrary to Hypotheses 7 and 8, attachment anxiety and avoidance did not moderate the effects of perceived partner phubbing on daily reports of anger and frustration with partner phubbing perceptions. Although participants overall reported more anger on days when phubbing was high, this did not differ by attachment patterns, suggesting it is frustrating and irritating to couple members in general. Mikulincer and Shaver (2005) propose that in response to negative partner behaviors, secure individuals experience functional anger, anxious individuals experience dysfunctional anger, whereas avoidant individuals experience suppressed anger. Our measures were not able to differentiate between these different types of anger, which may explain the lack of a moderating effect of attachment dimensions. This is a direction for future research.

Contrary to Hypothesis 5, anxiety did not moderate the effects of phubbing on relationship satisfaction. This was consistent with the dyadic daily diary results of Carnelley et al. (2023), but not with results obtained from cross‐sectional studies (e.g., Mosley and Parker 2023; Roberts and David 2016). As suggested earlier, attachment may have stronger effects on self‐reports of global behaviors rather than on daily behavioral reports due to the former being more susceptible to memory biases consistent with relational schemas. At least in daily reports, perceived partner phubbing seems particularly problematic for how those with high attachment anxiety feel about the self, but not the relationship. The main effect of daily phubbing on lower relationship satisfaction suggests it may be problematic for most couples.

Contrary to Hypotheses 4 and 6 (respectively), attachment avoidance did not moderate the effects of perceived partner daily phubbing on lower personal well‐being or relationship satisfaction. The findings for relationship satisfaction are consistent with those of past research using both cross‐sectional methods (Mosley and Parker 2023) and daily diary methods (Carnelley et al. 2023). The findings for well‐being are novel and suggest those high in avoidance are relatively immune to daily perceived partner phubbing; this may be a demonstration of their deactivating emotion regulation strategy and their turning away from potential relationship threat. Future research might examine whether self‐reports of those high in avoidance mesh with other indicators of distress that are less susceptible to self‐report biases, such as heart‐rate or skin‐conductance level.

### 4.2 Daily Responses to Being Phubbed

We found that individuals higher in attachment anxiety reported significantly higher curiosity, resentment, and retaliation (confirming Hypothesis 9) in response to perceived partner phubbing. This is consistent with Mikulincer and Shaver's (Mikulincer and Shaver 2016) predictions that attachment anxiety is associated with resentment in response to the partner's transgressions. However, we did not find the same effect of attachment avoidance on retaliation (contrary to Hypothesis 10). Attachment avoidance moderated the effect of daily perceived phubbing in predicting relational conflict. Although participants higher in attachment avoidance reported higher conflict on days when they perceived their partner as phubbing them more, they did this less so than did those low in attachment avoidance. The items that assessed conflict involved confronting the partner about their phone use—something that a highly avoidant person would feel less comfortable doing.

### 4.3 Daily Motivations for Retaliation

We explored whether attachment influenced retaliation motives and found that individuals higher in attachment anxiety reported a higher need for support and approval from others as reasons to retaliate (i.e., to phub their partner in response to being phubbed). This suggests that when partners ignore them, highly attachment‐anxious individuals turn to others to get their attachment needs met. This is consistent with past research showing that those high in attachment anxiety have a high need for approval and support in general (Collins and Feeney 2004; Lopez 2001). We also found that attachment avoidance moderated the effect of perceived partner phubbing in predicting the need for approval. Participants higher in attachment avoidance reported a higher need for approval as a motive to retaliate on days when they perceived their partner as phubbing them more; however, participants lower in attachment avoidance did not. This was somewhat surprising. It would be useful to know what sort of approval they are seeking and in what domain. Perhaps they are engaging in self‐presentation rather than self‐disclosure to gain approval; this might be a less intimate form of approval. Alternatively, they might be seeking approval for an autonomous or mastery activity. Future research might examine what individuals are doing when they retaliate—are they texting others, posting on social media in bids for attention, or simply reading or passively engaging with content? And do these activities differ by attachment styles? These are avenues for future work.

## 5 Strengths, Limitations, and Future Directions

The present study had several strengths: it was a diary study so it was able to capture daily responses to perceived phubbing rather than relying on retrospective reports of typical responses to perceived phubbing, and therefore it was less susceptible to biased reporting. The study was sufficiently powered to investigate the hypotheses which were pre‐registered. We examined novel, theoretically driven hypotheses about the role of attachment in responses to phubbing, thus contributing to our understanding of this potential relationship threat. Future research should investigate these processes over a longer time period to see whether even relatively secure participants report negative reactions to perceived partner phubbing if it is seen as frequent. Perhaps if phubbing occurs frequently over a longer time span, secure individuals will engage in constructive discussion of this behavior and set ground rules for technology use during couple interactions.

In addition, the study had some limitations. Although the study was sufficiently powered to detect small‐to‐medium effects, the interaction effects were often smaller than the planned effect size. Because we did not preregister one, we did not apply a Bonferroni correction post hoc. This may have resulted in some findings being significant by chance, and thus our findings should be replicated in future research. The study focused on self‐reports which may be subject to memory and social desirability biases. It would be useful to know whether psychophysiological indicators of emotion, such as heart rate and skin conductance level, are impacted by perceived phubbing and whether psychophysiological measures coalesce with self‐report measures of emotions, like anger, anxiety, and depression. A further limitation was that we did not test causal processes. Future directions include examining internal mediating mechanisms, couple interactions, and behavioral responses to phubbing, as well as manipulating partner phubbing to test causation. This data focuses on perceptions of partner phubbing and does not contain partner assessments of enacted phubbing. Future research might examine in a dyadic dataset how closely aligned reports of actor perceived phubbing and partner enacted phubbing are for people with different levels of attachment anxiety and avoidance. Although the sample was large, it was not very diverse in terms of gender (mostly female) and sexual orientation (mostly heterosexual). Future research should try to replicate these findings using more diverse samples (e.g., LGBTQ+), with varied relationship length and commitment levels, and from different countries.

## 6 Conclusion

In a diary study of daily perceived partner phubbing in a sample of couple members, we found the following results. Participants higher in attachment anxiety reported lower self‐esteem and higher depressed mood on days when they perceived their partner as phubbing them more; however, their relationship satisfaction was not impacted. Individuals higher (versus lower) in attachment anxiety also reported more curiosity, resentment, and retaliation in response to perceived partner phubbing. Although participants higher in attachment avoidance reported higher conflict on days when they perceived their partner as phubbing them more, they did this less so than those low in attachment avoidance. When retaliating, individuals higher in attachment anxiety did so in order to seek support and approval from others. Similarly, when retaliating, individuals higher in attachment avoidance did so in order to gain approval from others. These results add to our understanding of how adult attachment patterns influence couple interactions in the modern world of technology use. Interventions to improve couple functioning might focus on perceptions of partner phubbing and how retaliation may lead to a downward spiral of poor interactions. These may be particularly important for those who are high in attachment anxiety.

## Author Contributions

**Katherine B. Carnelley:** conceptualization, methodology, writing – review and editing, supervision. **Laura M. Vowels:** data analysis, writing – original draft and editing. **Claire M. Hart:** conceptualization, methodology, writing – review and editing, supervision. **Tessa Thejas Thomas:** data collection, writing – editing.

## Ethics Statement

The University of Southampton Faculty Ethics Committee approved the study, approval number ERGO 62732.A2. Participants gave written informed consent.

## Conflicts of Interest

The authors declare no conflicts of interest.

## Data Availability

All materials, including data and R code, can be found on the OSF project page: https://osf.io/ts6gw/?view_only=f4f774dbc6bc4de78eb7677c65dc4ab6. For the purposes of open access, the authors have applied a Creative Commons Attribution (CC BY) license to any author accepted manuscript version arising from this submission.

## Endnotes

^1^
In (Thomas et al. 2022), we report results for a subsample of the current sample (*N* = 75). These analyses focused on the effects of phubbing on personal and relational well‐being and retaliation motives; they do not focus on the effects of adult attachment patterns. The results presented in (Thomas et al. 2022) were sufficiently statistically powered (power = .80, *α* = .05), therefore we chose to publish them. Later we were able to acquire further funding to collect a larger sample and thus test the moderator effects of attachment. In subsequent footnotes, we mention when the current results (*N* = 196) replicate those with the smaller sample, however it is important to note that none of the current hypotheses were tested in the prior publication.

^2^
Narcissistic traits were assessed; results for narcissism will be reported in a separate paper.

^3^
We reconducted the analyses controlling for age, gender (men versus women), occupation (full‐time versus not), and working from home versus not. We found that everything that was significant before adding the covariates was still significant. Because of this, and for simplicity, we do not report the results with covariates included here, but they are available on the OSF. For transparency, we note that the covariates generally did not show significant associations. We note that the gender imbalance may not have provided sufficient power to detect gender effects.

^4^
These results are the same as those reported in (Thomas et al. 2022) based on the smaller sample, except that results for anxious mood were nonsignificant in the smaller sample.

^5^
These results are the same as those reported in (Thomas et al. 2022) based on the smaller sample, except that results for conflict and ignore were nonsignificant in the smaller sample.

^6^
These results are the same as those reported in (Thomas et al. 2022) based on the smaller sample, except that results for boredom were nonsignificant in the smaller sample.
